# An Integrative and Conjugative Element (ICE) Found in *Shewanella halifaxensis* Isolated from Marine Fish Intestine May Connect Genetic Materials between Human and Marine Environments

**DOI:** 10.1264/jsme2.ME22038

**Published:** 2022-09-02

**Authors:** Yuta Sugimoto, Aya Kadoya, Satoru Suzuki

**Affiliations:** 1 Center for Marine Environmental Studies, Ehime University, Matsuyama, Ehime, 790–8577 Japan; 2 Graduate School of Science and Engineering, Ehime University, Matsuyama, Ehime, 790–8577 Japan

**Keywords:** integrative and conjugative element, resistance, marine bacteria, plasmid

## Abstract

Integrative and conjugative elements (ICEs) play a role in the horizontal transfer of antibiotic resistance genes (ARGs). We herein report an ICE from *Shewanella halifaxensis* isolated from fish intestine with a similar structure to both a clinical bacterial ICE and marine bacterial plasmid. The ICE was designated ICE*Sha*Jpn1, a member of the SXT/R391 family of ICEs (SRIs). ICE*Sha*Jpn1 has a common core structure with SRIs of clinical and fish origins and an ARG cassette with the pAQU1 plasmid of *Photobacterium damselae* subsp. *damselae*, suggesting that the common core of SRIs is widely distributed and ARG cassettes are collected from regional bacteria.

When antibiotic resistance genes (ARGs) are transferred among different bacterial species, horizontal gene transfer (HGT) is mediated by various mobile genetic elements (MGEs). Integrative conjugative elements (ICEs) (Partridge *et al.*, 2018) are one of the MGEs, encoding the machinery for the conjugation process of HGT from the donor chromosome into the new host chromosome (Wozniak and Waldor, 2010). A recent study suggested that 50% of bacterial genomes harbor ICEs, which contribute to HGT within and across bacterial taxa (Kaufman, J.H., *et al.*, 2020. Integrative and conjugative elements (ICE) and associated cargo genes within and across hundreds of bacterial genera. *bioRxiv* https://doi.org/10.1101/2020.04.07.030320).

Among ICEs, SXT/R391-family ICEs (SRIs) form a large group of MGEs listed in the ICEBerg (http://db-mml.sjtu.edu.cn/ICEberg/) database (Bi *et al.*, 2012), which was updated in 2018 (https://bioinfo-mml.sjtu.edu.cn/ICEberg2/index.php). The bacterial hosts of SRIs include a number of bacteria isolated from human, animal, and water environments (Burrus *et al.*, 2006; Pembroke and Piterina, 2006; Poulin-Laprade *et al.*, 2015), suggesting that SRIs have a broad host range, including aquatic bacteria, and play a role in the dissemination of ARGs across various bacterial communities in aquatic environments. Marin *et al.* (2014) reported that the same ARGs were shared among different SRIs in pathogens. SRIs are expected to be natural reservoirs of ARGs and contribute to HGT among bacteria in different environments.

Common ARGs are widely distributed among culturable and non-culturable bacteria in marine environments (Rahman *et al.*, 2008; Suzuki *et al.*, 2013, 2019). A new macrolide resistance gene set, *mef*(C)-*mph*(G) (Nonaka *et al.*, 2012, 2014, 2015) was recently detected on MGEs of various sizes (Sugimoto *et al.*, 2017). This gene set was originally identified in plasmid pAQU1, which is a multi-resistance plasmid of *Photobacterium damselae* subsp. *damselae* strain 04Ya311 (Nonaka *et al.*, 2012). We hypothesized that the shared *mef*(C)-*mph*(G) gene set is conveyed not only on plasmids, but also on chromosomal elements. Marine environmental bacteria may act as reservoirs of transferrable ARGs with coding on various MGEs.

We isolated *Shewanella halifaxensis* strain 6JANF4-E-4, a *mef*(C)-*mph*(G)-possessing bacterium from the intestine of red seabream (*Pagrus major*). The draft genome sequence was elucidated by PacBio RSII and assembled using single-mole­cular real-time portal ana­lysis (SMRT) software version 2.3 (Pacific Biosciences) (Sugimoto *et al.*, 2018). Genetic annotation and sequence-based comparisons were performed in the present study by Rapid Annotations using Subsystems Technology (RAST) and SEED Viewer (Aziz *et al.*, 2008; Overbeek *et al.*, 2005). We designated the new ICE as ICE*Sha*Jpn1 according to international nomenclature (Burrus *et al.*, 2006). A BLASTN search was used to align the ICE*Sha*Jpn1 part to the sequences of SRIs in the GenBank database. Based on BLASTN results, 14 SRIs with different scores and origins were selected to con­struct‍ ‍a‍ ‍phylogenetic tree. The draft genome sequence of 6JANF4-E-4 was deposited in DDBJ/EMBL/GenBank under Accession Numbers BFBQ01000001 to BFBQ01000005 (Sugimoto *et al.*, 2018), where the ICE region is identified on BFBQ01000004. The locus tag is shown in Fig. S1.

To date, ICEs have been detected in a wide variety of‍ ‍*Proteobacteria*, such as *Vibrio*, *Photobacterium*, *Providencia*, *Proteus*, *Alteromonas*, *Marinomonas*, and *Shewanella* (Pembroke and Piterina, 2006; Fang *et al.*, 2018). *Shewanella* species are ubiquitous inhabitants of marine environments, while some are pathogens of fish and humans (LPSN, https://www.bacterio.net/genus/shewanella) (Hau and Gralnick, 2007). This finding suggests that ICE-possessing *Shewanella* spp. in various environments are universal carriers of ARGs.

ICE*Sha*Jpn1 of 6JANF4-E-4 harbored 95 coding sequences (CDS). Wozniak *et al.* (2009) reported that SRIs consisted of 52 core genes along with additional variable regions. The core genes involved functions including excision and integration, DNA repair, transfer, DNA recombination, and regulation. ICE*Sha*Jpn1 possessed all of these core genes, along with a unique region containing five putative ARGs with sequence identity to *floR*, *mph*(G), *mef*(C), *sul2*, and a β-lactamase gene in that order (Fig. S1). Among the five genes, the *mef*(C)-*mph*(G) set was oriented in the opposite direction to other ARGs. Therefore, *mef*(C)-*mph*(G) may be co-transcribed in this order, as has been reported for other MGEs (Sugimoto *et al.*, 2017).

The alignment of the nucleotide sequence of ICE*Sha*Jpn1 with other SRIs showed 73 to 81% sequence identity. Based‍ ‍on a phylogenetic tree constructed using the nucle­otide sequences of 52 core genes, ICE*Sha*Jpn1 showed the closest relationship to ICE*Pda*Spa1, an SRI carried by *Photobacterium damselae* subsp. *piscicida* isolated from a diseased fish in Spain (Juíz-Río *et al.*, 2005) (Fig. 1). Other ICEs from *Shewanella* spp. (ICE*Spu*CHN110003, ICE*Sh*95, ICE*Spu*PO1, and ICE*Sh*392) showed lower identities. This result suggested that SRI sequences do not clearly correlate with the taxon of the bacterial host.

Although ICE*Sha*Jpn1, ICE*Pda*Spa1, and SXT^MO10^ have distinct histories (Osorio *et al.*, 2008; Taviani *et al.*, 2009; Sugimoto *et al.*, 2017), these three MGEs were assigned to the same cluster based on sequence similarities. On the other hand, SXT^MO10^ and ICE*Vch*CHNAHV1003 (Wang *et al.*, 2016), both identified in *Vibrio cholerae* from patients in India, were assigned to different clusters. These findings indicate that various SRIs are spread across wide areas and are not restricted to specific geographic areas or genera. Our phylogenetic tree also showed that the region of origin and year of isolation widely varied among SRIs, again suggesting that various SRIs are distributed across wide areas and various hosts.

Four shared SRI core genes are shown at the top of Fig. 2A. ICE*Pda*Spa1 and SXT^MO10^ are assigned to the same cluster as ICE*Sha*Jpn1, and ICE*Vch*Ban9 belongs to the next closest cluster, but has higher identity (81%). Core genes include those encoding transposition and conjugation machineries. Comparisons among the four SRIs suggested the presence of “variable regions” (I~IV) and “hot spots” (HS1~5) (Fig. 2A). Variable region III is an ARG cassette region harboring a gene (*tnpA*) encoding a transposase of the Tn*3* family, and in which most ARGs were located. ICE hot spots are considered to be areas for the acquisition of new DNA (Beaber *et al.*, 2002). The variable ARG cassette region of ICE*Sha*Jpn1 showed sequence identities of between 38 and 70% with other ICEs (Fig. 2B). *floR*, *strA-B*, and *sul2* are reportedly spread among the SRIs of *Vibrio cholerae* O1 strains (Marin *et al.*, 2014). SXT^MO10^ harbors *floR*, *strA-B*, and *sul2*; ICE*Vch*Ban9 harbors *floR*, *tetA-R*, *strA*, *strA-B*, and *sul2*. ICE*Sha*Jpn1 harbors *floR* and *sul2*; however, *strA-B* were replaced by *mef*(C)-*mph*(G) (Fig. 2B). This ARG pattern is the same as that observed on plasmid pAQU1 isolated from seawater in Japan (Nonaka *et al.*, 2012). This variable region of ICE*Sha*Jpn1 has high identity (86%) to pAQU1 sequences, particularly in the *floR-sul2* region, which exhibits 96% identity across 5915 bp (Fig. 2B). This sequence is shared not only with pAQU1, but also with other related plasmids that have been found in marine environments in Japan (Nonaka *et al.*, 2014). Similar ARG cassettes conveyed by different MGEs are presumably disseminated within a limited geographical area. Additionally, the ARG cassette region of ICE*Sha*Jpn1 harbored the duplicated *tnpB* gene shared with other SRIs and pAQU1. Previous studies suggested that IS*91* elements are ARG capture and movement systems that are present on plasmids and chromosomes (Toleman *et al.*, 2006; Toleman and Walsh, 2008). These elements may contribute to the assembly of ARGs from various SRIs and plasmids. Hochhut and Waldor (1999) reported that an SXT element was inserted into the *prf*C gene; however, our 6JANF4-E-4 does not have an identical sequence to *prf*C on the genome.

The conjugative transfer of ICE*Sha*Jpn1 from the 6JANF4-E-4 strain to *Escherichia coli* strain JW0452 was performed according to Neela *et al.* (2009). The conjugation frequency was (7.8±2.7)×10^–5^. Antibiotic susceptibility tests were performed on 6JANF4-E-4, *E. coli* JW0452, and transconjugant *E. coli* TJ6JANF4-E-4. 6JANF4-E-4 was incubated at 25°C overnight in 1‍ ‍mL of LB plus 2% NaCl; *E. coli* strains were incubated at 37°C overnight in 1‍ ‍mL of LB. Cell density was adjusted to McFarland optical density No. 0.5 with phosphate-buffered saline. An aliquot (100‍ ‍μL) of the cell suspension was spread on Mueller-Hinton agar (*E. coli*) or Mueller-Hinton plus 2% NaCl agar (6JANF4-E-4) as previously reported (Kawanishi *et al.*, 2005; Sugimoto *et al.*, 2017). Etest strips (bioMérieux) were then placed on each plate. MICs were assessed for erythromycin (EM), azithromycin (AZ), sulfamethoxazole (SX), sulfamethoxazole/trimethoprim (TS), chloramphenicol (CL), ampicillin (AM), imipenem (IP), cefotaxime (CT), and aztreonam (AT) by inspecting for cell growth after an overnight incubation at 25°C for 6JANF4-E-4 and at 37°C for *E. coli*. To confirm the completion of conjugation, putative conjugants were screened by PCR for the presence of the *traI* gene, which is located on ICE*Sha*Jpn1. PCR conditions were the same as those previously reported (Nonaka *et al.*, 2014). Product bands were obtained from strains 6JANF4-E-4 and TJ6JANF4-E-4, but not from *E. coli* JW0452 (Fig. S2).

The MICs of four types of antibiotics are shown in Table S1. The parent strain, 6JANF4-E-4, was resistant to EM, AZ, and AM (with MICs of >256, 8, and >256‍ ‍μg mL^–1^, respectively), and susceptible to CL, SX, TS, IP, CT, and AT, even though 6JANF4-E-4 harbored ARGs expected to encode resistance against florfenicol/chloramphenicol (*floR*), sulfonamide (*sul2*), and ß-lactam (encoding a putative ß-lactamase). The low MIC to sulfonamides may reflect the presence of a 21-bp deletion in *sul2* (relative to homologous genes) that is predicted to impair the activity of the encoded protein. On the other hand, the observed susceptibility to ß-lactams other than AM may reflect the substrate specificity of the ICE*Sha*Jpn1-encoded ß-lactamase for AM (Lee *et al.*, 2005). Regarding macrolide resistance genes, a previous study reported that the macrolide resistance genes harbored by the genomic ICEP*mu*1 of *Pasteurella multocida* (Michael *et al.*, 2012) were derived from a plasmid. Similarly, *mef*(C)-*mph*(G) harbored by ICE*Sha*Jpn1 were shared with pAQU1, suggesting some relationship between ICE*Sha*Jpn1 and pAQU1 within the same geographical area. To assess the role of SRIs in the environment, the relationships between resistance genes and SRIs in the environment warrant further study.

In conclusion, a novel SRI identified in an isolate of *S. halifaxensis* harbored an ARG cassette shared with plasmid pAQU1. The combination between a common SRI core structure and unique ARG cassette suggests the dissemination of ARGs in various environments by SRIs. These properties may contribute to the dissemination of ARGs between environmental and clinical SRIs. Further research is expected to clarify the mechanisms of evolution of ARGs across multiple MGEs, which will assist in risk assessments of the spread of antibiotic-resistant bacteria in aquatic environments. A more detailed understanding of gene migration between marine and human bacteria needs to be considered as part of the “One Health” concept (G7 Presidency, 2015).

## Funding

This work was supported by KAKENHI (grant numbers 22241014, 22254001, 25257402, 16H01782, and 20H00633), JSPS, Japan.

## Citation

Sugimoto, Y., Kadoya, A., and Suzuki, S. (2022) An Integrative and Conjugative Element (ICE) Found in *Shewanella halifaxensis* Isolated from Marine Fish Intestine May Connect Genetic Materials between Human and Marine Environments. *Microbes Environ ***37**: ME22038.

https://doi.org/10.1264/jsme2.ME22038

## Supplementary Material

Supplementary Material

## Figures and Tables

**Fig. 1.**
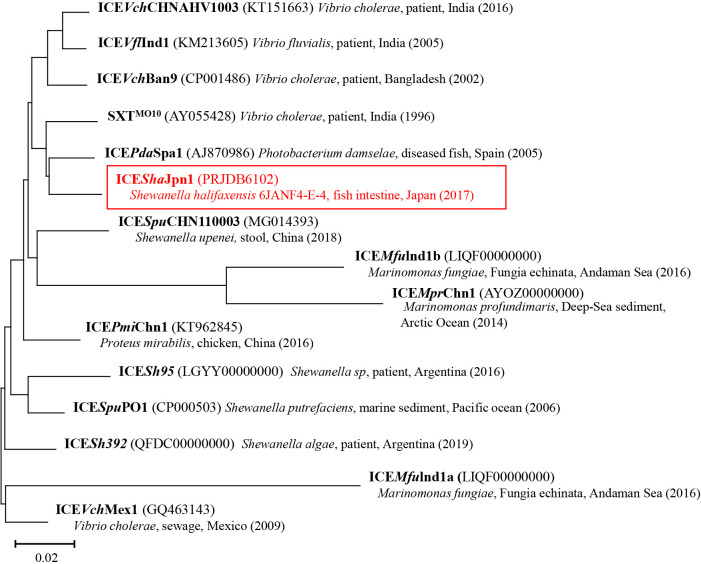
The phylogenetic relationship of elements based on 52 genes of the SRI core region. The tree was constructed using the neighbor-joining method with the tree configuration generated using 1,000 bootstrap trials. The scale bar indicates 0.02 fixed nucleotide substitutions per sequence position. The accession numbers of sequences used in this ana­lysis are shown in parentheses.

**Fig. 2.**
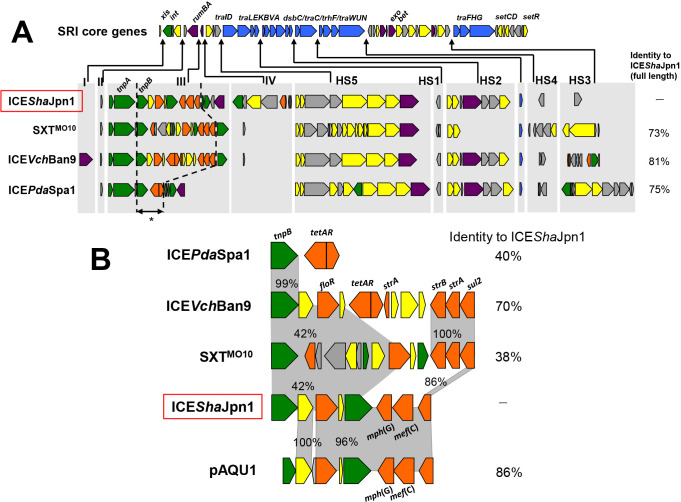
A; SRI core genes and variable regions of four SRIs. The arrow indicates the sites of insertions of variable regions (I-IV) and five hotspots (HS1-5) relative to other SRIs. Identity (%) to ICE*Sha*Jpn1 is obtained from nucleotide sequences within this region. The variable ARG region (indicated with an asterisk) is expanded in B. B; Sequence alignment of nucleotides encoded by the ARG cassettes of SRIs and plasmid pAQU1. Identity (%) to ICE*Sha*Jpn1 is based on the ARG cassette region’s nucleotide sequence in this figure. Percent number in gray shading shows the identity of the indicated part between each element. Gene colors indicate the putative function of the encoded proteins: green, transposition; orange, ARG; yellow, other.
