# Supplementary material for: An Integrative and Conjugative Element (ICE) Found in Shewanella halifaxensis Isolated from Marine Fish Intestine May Connect Genetic Materials between Human and Marine Environments
Source: Microbes Environ. 2022 Sep 2;37(3):ME22038. doi: 10.1264/jsme2.ME22038 (PMC9530723; doi:10.1264/jsme2.ME22038)
Supplement: Supplementary file 1 — Supplementary Material [file 37_22038_s1.pdf]

Supplement materials (Sugimoto Y et al.)

Table S1. The MICs of 6JANF4-E-4, JW0452 and transconjugant TJ6JANF4-E-4.

	MIC (μg/mL)		
**Antibiotic**	**6JANF4-E-4**	**JW0452**	**TJ6JANF4-E-4**
Erythromycin (EM)	>256	1.5	>256
Azithromycin (AZ)	8	0.75	3
Sulfamethoxazole (SX)	4	6	6
Sulfamethoxazole + Trimethoprim (TS)	0.016	0.006	0.006
Chloramphenicol (CL)	1.5	0.5	16
ß-lactam			
Ampicillin (AM)	>256	0.5	>256
Imipenem (IP)	0.25	0.19	0.19
Cefotaxime (CT)	0.002	0.08	0.08
Aztreonam (AT)	0.47	0.32	0.32

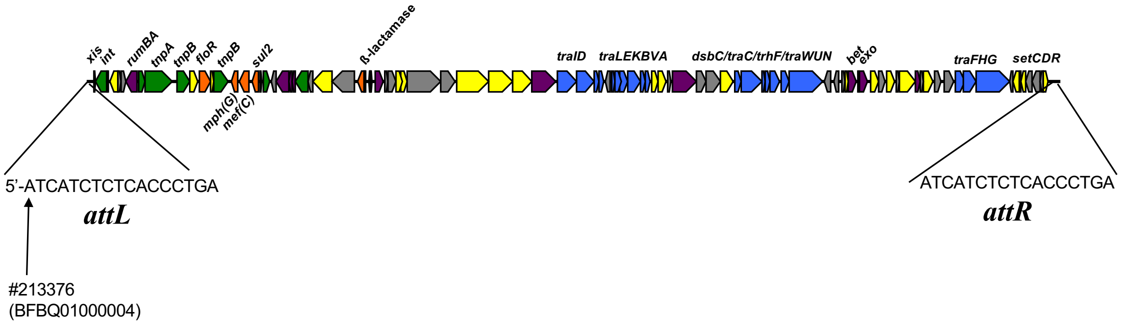

Fig. S1. Genetic structure of ICEShaJpn1 flanked by 17-bp *attL* and *attR* sites. Total length, 107,133 bp. Locus tag is indicated at 5'-end of *attL* with nucleotide number of the sequence data (accession number BFBQ01000004). The genes involved in SRI maintenance are as follows: excision and integration (*xis* and *int*), DNA repair (*rumBA*), gene transfer (*tra* genes), DNA recombination (*bet* and *exo*), and regulation (*setCDR*). Gene colors indicate the putative function of the encoded proteins: green, transposition; blue, conjugation; purple, DNA processing; orange, ARGs; yellow, other; gray, unknown.

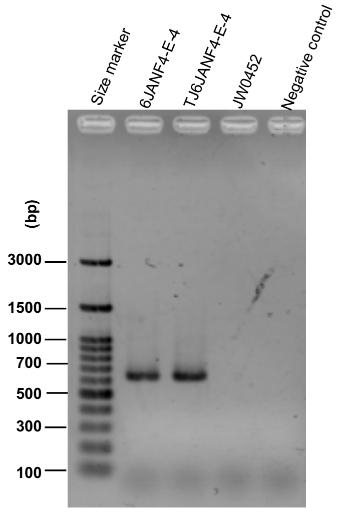

Fig. S2. PCR product for *traI* in *Shewanella halifaxensis* strain 6JANF4-E-4, transconjugant *E. coli* TJ6JANF4-E-4 and *E. coli* JW0452. Negative control does not contain template. The product band size is 645 bp. Size marker is FasGene^{TM} DNA Ladder (NIPPON Genetics).
